# Applying Molecular Dynamics Simulations to Identify Rarely Sampled Ligand-bound Conformational States of Undecaprenyl Pyrophosphate Synthase, an Antibacterial Target

**DOI:** 10.1111/j.1747-0285.2011.01101.x

**Published:** 2011-06

**Authors:** William Sinko, César de Oliveira, Sarah Williams, Adam Van Wynsberghe, Jacob D Durrant, Rong Cao, Eric Oldfield, J Andrew McCammon

**Affiliations:** 1 Department of Chemistry & Biochemistry, and NSF Center for Theoretical Biological Physics, University of California San DiegoLa Jolla, CA 92093-0365, USA; 2 Department of Pharmacology, and NSF Center for Theoretical Biological Physics, University of California San DiegoLa Jolla, CA 92093-0365, USA; 3 Biomedical Sciences Program, University of California San DiegoLa Jolla, CA 92093-0365, USA; 4 Howard Hughes Medical Institute, University of California San DiegoLa Jolla, CA 92093-0365, USA; 5 Department of Chemistry, Hamilton CollegeClinton, NY 13323, USA; 6 Department of Chemistry, University of Illinois at Urbana–Champaign, 600 South Mathews Avenue, Urbana, IL 61801, USA

**Keywords:** active conformations, bisphosphonates, computer aided drug design, docking, inhibitors, molecular dynamics, undecaprenyl pyrophosphate synthase

## Abstract

Undecaprenyl pyrophosphate synthase is a *cis*-prenyltransferase enzyme, which is required for cell wall biosynthesis in bacteria. Undecaprenyl pyrophosphate synthase is an attractive target for antimicrobial therapy. We performed long molecular dynamics simulations and docking studies on undecaprenyl pyrophosphate synthase to investigate its dynamic behavior and the influence of protein flexibility on the design of undecaprenyl pyrophosphate synthase inhibitors. We also describe the first X-ray crystallographic structure of *Escherichia coli apo*-undecaprenyl pyrophosphate synthase. The molecular dynamics simulations indicate that undecaprenyl pyrophosphate synthase is a highly flexible protein, with mobile binding pockets in the active site. By carrying out docking studies with experimentally validated undecaprenyl pyrophosphate synthase inhibitors using high- and low-populated conformational states extracted from the molecular dynamics simulations, we show that structurally dissimilar compounds can bind preferentially to different and rarely sampled conformational states. By performing structural analyses on the newly obtained *apo*-undecaprenyl pyrophosphate synthase and other crystal structures previously published, we show that the changes observed during the molecular dynamics simulation are very similar to those seen in the crystal structures obtained in the presence or absence of ligands. We believe that this is the first time that a rare ‘expanded pocket’ state, key to drug design and verified by crystallography, has been extracted from a molecular dynamics simulation.

The evolutionary pressure of antibiotics has created drug-resistant strains in most species, creating the need for new drugs that inhibit targets not previously exploited by clinicians (1,2). Inhibition of enzymes involved in isoprenoid biosynthesis is a promising new target area as many of these enzymes are not present in humans. For example, the enzymes that comprise the non-mevalonate pathway leading to the biosynthesis of isopentenyl diphosphate (IPP) and dimethylallyl diphosphate are not present in humans, which use the mevalonate pathway (3). The enzymes involved in the later stages of isoprenoid biosynthesis, such as undecaprenyl diphosphate synthase (UPPS), are also targets of interest in drug discovery. Undecaprenyl pyrophosphate synthase is a *cis*-prenyl transferase and is responsible for the condensation of eight molecules of IPP with farnesyl diphosphate to form the C_55_ species, undecaprenyl diphosphate (Scheme 1).

Following the condensation, undecaprenyl diphosphate is converted to Lipid I, Lipid II, and lastly, to bacterial cell wall peptidoglycans. Many antibiotics (e.g. the β-lactams penicillin and methicillin; cephalosporins; and glycopeptides such as vancomycin and teicoplanin) inhibit cell wall biosynthesis. Undecaprenyl pyrophosphate synthase is an attractive target in e.g. *Staphylococcus aureus* as there is only modest homology (34% identity, 54% similarity, E-value = 8e^−38^) to the human *cis*-prenyl transferase, dehydrodolicol diphosphate synthase.

Numerous X-ray crystal structures of UPPS have been solved with the farnesyl pyrophosphate (FPP) and IPP substrates or inhibitors bound (4–8). Comparison of these crystal structures reveals that UPPS is a highly flexible protein system, showing very mobile binding pockets in the active site region. The catalytic site is composed of four binding pockets (8) (Figure S1A), and it is responsible for accommodating the large substrates, FPP and IPP, and for transferring the phosphate groups and metal ions between the two substrates during the condensation reaction (7). However, the available crystallographic data have proven insufficient for a successful structure-based drug design campaign. There have been several reports of high-throughput screens (HTS) against UPPS. In one study, several hits were claimed, but the structures were not disclosed (9). In a second experimental screen, no hits were reported (10), but in a third screen workers at Novartis reported several promising leads; tetramic and tetronic acids as well as dihydropyridine-2-ones (11,12). On the *in silico* or virtual HTS front, based on a *Helicobacter pylori* crystal structure, Kuo *et al.* (13). reported two hits (with IC_50_ values in the ∼70–500 μm range) against *H. pylori* UPPS and *E. coli* UPPS, with some selectivity against the *H. pylori* protein (13). In earlier work, we have characterized 29 bisphosphonate compounds and five co-crystal structures (8). These bisphosphonates and the Novartis Peukert *et al.* (11) substituted tetramic acid and dihydropyridin-2-one-3-carboxamide structures are shown in Chart 1.

In this work, we use experimentally validated compounds (Chart 1) to correlate our *in silico* data with known experimental results. In addition, long molecular dynamics (MD) simulations of the *apo* form of the enzyme, starting from an inhibited conformation, and docking studies were performed on UPPS to investigate its dynamic behavior and the influence of protein flexibility on the design of UPPS inhibitors. We were able to identify ‘*active conformational states’* of the *apo* form of UPPS that recognize different classes of known inhibitor molecules, a discovery that may be useful in virtual screening efforts. We used a new descriptor, active site volume, to find rare pocket conformations. In addition, we have crystallized *E. coli* UPPS with the flexible loop to further investigate the large pocket-size fluctuation in our MD simulations.

## Experimental Section

### Computational details

The crystal structure of the UPPS enzyme in complex with the bisphosphonate, BPH-629 (PDB ID 2E98), was used to build the models for the MD simulations (8). All bisphosphonate ligands were removed from the active sites of each monomer, and the protein system was simulated in the *apo* state. Chain B of the UPPS homodimer lacks crystallographic information for residues 73–82, which were modeled using MODLOOP (14,15). The protonation states of the residues were determined using the propka program (16,17), with special attention paid to His43. Residue His43 is well positioned to hydrogen bond the diphosphate groups of the bound ligands, and it is thought to not only play an important role in the binding process of the natural substrates, but also to be of key importance to the catalytic activity of the enzyme (18). Owing to the importance of this residue, the effect of different protonation states of His43 on the dynamics of UPPS was investigated through two MD simulations: one where HIS43 is singly protonated (HID43), and the other where HIS43 is doubly protonated (HIP43).

In both simulations, TIP3P water molecules were used as the solvent model in a truncated octahedron. Water molecules and counter ions, Cl^−^, were added to solvate the structure and neutralize the total charge of the protein using the amber program xLeap. Simulations were performed using the sander. MPI module of AMBER 10, the AMBER ff99SB forcefield, and Particle Mesh Ewald to describe the electrostatic interactions (19). Temperature control was achieved with the weak-coupling algorithm, and pressure control was accomplished via isotropic position scaling (20). Energy minimization of the solvated system was performed with an initial 1500 steps of steepest descent, followed by 500 steps of conjugate gradient minimization. To bring the system to the correct density and allow proper re-orientation of the water molecules, an MD simulation of 100 ps was performed in the NPT ensemble where the protein was fixed and only the water molecules were allowed to move freely. Following this, the entire system was heated from 0 K to 300 K over 500 ps of MD simulation in the NVT ensemble. To ensure complete equilibration of the system at 300 K, 200 ps of MD simulation was performed. All analyses were carried out on an additional MD simulation of 85 ns, in which the NVT ensemble was applied. All simulations were stable as shown by root mean squared deviation plots (Figure S2). The setup, equilibration, and production protocols were applied to both HID and HIP systems.

**Figure 1:**
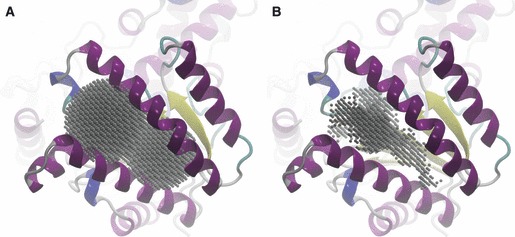
Grid points used to calculate pocket volumes. (A) Points spaced 1 Å apart were positioned along a grid that encompassed the UPPS IPP-binding pocket. (B) Those grid points near protein atoms were removed, leaving only points within the active site, from which the volume was calculated.

To calculate the volume of the active site of UPPS, frames were extracted from the MD simulations every 10 ps and aligned. The povme software was then used to define a volume that encompassed the active sites by taking into account carefully positioned spheres of 10 Å radii, manually centered in the active site region (21). Points spaced 1 Å apart were positioned along a grid within this defined volume (Figure 1). The same positioning of grid points was used for all structures so volumes would be comparable. For each frame, a hydrogen atom was positioned at each grid point, and wherever van der Waals clashes with protein atoms occurred, the point was removed. Small, isolated clusters of points were likewise removed. The remaining points were judged to be contained within the active site (Figure 1). As the points were originally spaced 1 Å apart, each point corresponded to a region of space 1 Å^3^ in volume, allowing the volume of the binding pocket to be easily calculated. The pocket volume of the *holo* [PDB ID: 2E98, 2E99 (11) 1X06, 1X07, 1X08, 1X09 (7)] and the *apo* crystal structures were likewise calculated. Crystallographic waters and co-crystallized ligands were removed. While other crystal structures of *E. coli* UPPS have been solved, they lack some residues in the active site, and therefore their volume could not be accurately calculated.

### Crystallization and structure determination of apo *E. coli* UPPS

The *E. coli* UPPS enzymes were expressed and purified as described previously (22). *Apo*-UPPS crystals were obtained as described previously, with modifications (11). Briefly, 1 μL UPPS protein solution was mixed with 1 μL of mother liquor (5% PEG4K), then equilibrated with 500 μL of mother liquor at room temperature by using hanging drop evaporation. X-ray diffraction data were collected at the Life Science Colloborative Access Team (LS-CAT) 21-ID-F at the Advanced Photon Source of Argonne National Laboratory. Diffraction data were processed and scaled by using the program HKL2000 (HKL Research Inc., Charlottesville, VA, USA) (23). The statistics for data collection are included in Table 1. For structure determination, a molecular replacement calculation was carried out by using a model prepared from the BPH-629 UPPS structure (PDB ID 2E98) with ligands and solvent removed. The 2Fo−Fc difference Fourier map showed clear electron densities for most amino acid residues, including those in the flexible loop in chain A. Iterative rounds of refinement using Refmac (24,25) and rebuilding of loop residues using Coot (26) were then carried out. Further refinement was performed in Refmac5 with the TLS parameters generated by the TLSMD server (27). The resulting final structure has an R-factor of ∼18.6% (R_free_ ∼ 22.4%). The refinement statistics are included in Table 1.

**Table 1:** Data collection and refinement statistics for undecaprenyl pyrophosphate synthase apo crystal (3QAS)

Data collection	
Space group	P2_1_2_1_2_1_
Unit cell dimension (Å)	
*a*, *b*, *c* (Å)	62.633, 68.762, 111.826
X-ray source	LS-CAS 21-ID-F
Wavelength (Å)	0.9787
Resolution (Å)	50–1.70 (1.73–1.70)
No. of reflection observed	385, 942
Unique	53 420 (2600)
Completeness (%)	97.5 (99.7)
*R-*merge	0.051 (0.400)
*I/σI*	51.2 (3.7)
Multiplicity	7.2 (5.8)
Refinement statistics	
Resolution range (Å)	50.0–1.70
*R*-work/*R*-free (%)	18.6/22.4
RMSD	
Bond lengths	0.026
Bond angles	2.115
No. of Protein atoms	3453
*B* average (Å^2^) of protein	28.77
Ramachandran plot statistics	
Residues in preferred regions (%)	95.3
Residues in allowed regions (%)	4.4
Residues in generously allowed regions (%)	0.3

## Results

### Docking of known UPPS inhibitors

**Figure 2:**
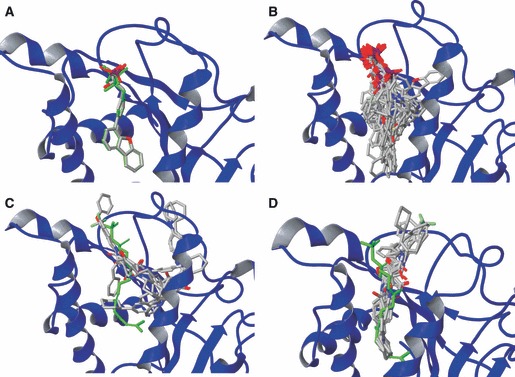
(A) Superposition of the docked (colored by atom type) and co-crystallized poses (green) of BPH-629 bound to the 2E98 crystal structure. (B) Docked poses of 29 BPH inhibitors into the 2E98 structure. (C) Docked poses of 1i, 1j, 4a, 4g, 4j, 4l, and 4m into the 2E98 structure. Ligand poses showed a very poor alignment with the substrate, farnesyl pyrophosphate (FPP) (shown in green). (D) Docked poses of 1i, 1j, 4a, 4g, 4j, 4l, and 4m into the fourth most populated MD-derived structure. All ligand poses showed good alignment with the substrate, FPP (shown in green).

In earlier work (8), we reported the X-ray crystal structures of five bisphosphonate compounds bound to *E. coli* UPPS. We found up to four distinct binding sites for BPH-629 (PDB 1D code 2E98). However, only Site 1 (Figure 2A) was occupied in all of the five bisphosphonate/UPPS structures reported, with ligand interactions involving ASP-26, ASN-28, ARG-39, HIS-43, ARG-51, ARG-77, PHE-89, ARG-102, and HIS-103 being common to all structures. On average, there were ∼14 bisphosphonate–protein interactions in Site 1, but only ∼9 for Sites 2–4, compared with 15 interactions (in Site 1) for the substrate analog, S-*thiolo*-farnesyl diphosphate (FSPP). This suggests that Site 1 might represent the strongest binding site for bisphosphonate UPPS inhibitors. To test this hypothesis further, we carried out a computational docking investigation using the Glide program^a^ (28,29).

The co-crystallized structure of UPPS and BPH-629 (PDB ID 2E98), after removal of ligands, was subjected to numerous docking calculations with the program GLIDE at the XP level. Four BPH-629 ligands are present in the active site of chain A of UPPS in the crystal structure, identifying four distinct binding sites. As shown in Figure 2, the docking calculation accurately reproduced the crystallographic poses of BPH-629 in the UPPS-active site. Although four binding sites were identified in the UPPS crystal structure, the poses of all bisphosphonate (BPH) ligands generated by GLIDE were located primarily in the first binding site (Figure 2B). These docking results are also in good agreement with the experimental observation that, in the crystal structure, ligands bind more tightly to the first binding site (8). To further validate our model, the 29 BPH ligands (Figure 2), with known potency against UPPS, were docked into the structure, and the estimated free energy of binding (Glide XP score) of each ligand was compared with its respective pIC_50_. When these docking results are compared with the enzyme inhibition pIC_50_ results (pIC_50_ = −log_10_ IC_50_ [M]), we find a correlation coefficient of −0.5 (Figure S3). The correlation coefficient is promising but not strong. This could be in part because of the many approximations inherent in docking software, or artifacts from crystallographic conditions including the high concentration of ligand in the medium (5 mm). The structural changes induced by the three other inhibitors occupying the active site, which were removed for docking, may also affect the correlation.

In recent work, Peukert *et al.* (11) described a class of potent and selective UPPS inhibitors. The scaffold from a known UPPS inhibitory compound was modified to create a small library of substituted tetramic acid and dihydropyridin-2-one-3-carboxamides. These compounds, inspired by the binding mode of FPP, possess two hydrogen bond acceptors and a hydrophobic group, which are important interaction sites. The proposed inhibitors showed sub-micromolar UPPS inhibition and antibacterial activity against Gram-positive bacteria. To investigate the binding mode of this class of inhibitors, inhibitors **1i**, **1j**, **4a**, **4g**, **4j**, **4l,** and **4m** (Figure 2) were docked into the crystal structure 2E98. These inhibitors bear no structural similarity with the BPH compounds, and they were designed to adopt a binding mode similar to the natural substrate, FPP. As displayed in Figure 2C, docking of these inhibitors into 2E98 generated unexpectedly poor results with unreasonable binding poses. The molecules were distributed over all four binding sites with none of the poses showing a good alignment with FPP. This raised the question: is the protein in a different conformation when it binds these non-bisphosphonate inhibitors?

### Identifying inhibitor-bound UPPS conformations from MD simulations

**Figure 3:**
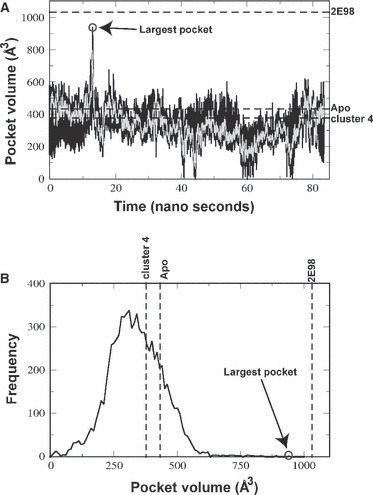
Volume distribution of the HIP43 undecaprenyl pyrophosphate synthase binding pocket 2E98 crystal structure and the *apo* crystal structure. (A) Volume of the binding pocket along the MD trajectory. The black line shows data taken every 10 ps, the overlayed gray line is the average over every 100ps. (B) Frequency at which different volumes of the pocket are sampled. The size of the bisphosphonate-bound crystal structure (2E98), the newly described *apo* crystal structure (*Apo*), and cluster 4 that docked tetramic acids well, are represented by labeled dashed lines in both graphs.

To investigate the dynamic behavior of UPPS, we performed clustering analysis of the MD trajectories of the HIP43 and HID43 systems. Each trajectory was fit to the alpha carbons of all UPPS subunit A residues, with the exception of the C and N-termini and the flexible segment containing residues 73–80. Clustering was performed by employing the Gromos method with RMS differences of a selection of active site residues (residues 23–51, 67–93, 96, 110, 141–145, 194, 204, 221–222) within gromacs version 3.3.1.^b^ To choose the appropriate cutoff radius, several cutoffs were investigated, resulting in a final cutoff of 1.8 Å for the HID43 simulation and a cutoff of 2.2 Å for the HIP43 simulation. The first five clusters represent more than 90% of the entire trajectory. Docking of the 29 BPH ligands into the five representative MD cluster structures did not show any improvement over the results from the crystal structure. The clustered structures demonstrate that the crystal conformation is not highly populated in the *apo* enzyme trajectory. It is worth noting that the crystal structure 2E98 contains four BPH-629 molecules in its active site because of the high inhibitor concentration used in the experiment (8). Thus, in this case, it is expected that induced fit effects are playing an important role in the binding process, promoting large conformational changes in the active site of the enzyme, expanding the volume from the unbound state. The active site of 2E98 is very large when compared with the most populated structures extracted from the MD simulations (1032 Å^3^ compared to an average of 332 Å^3^ volume in the MD simulations). Clustering analysis showed that all representative structures from the MD simulation displayed active sites with significantly decreased volumes when compared to the crystal structure. We believed that this highly open conformation could be favoring the binding of BPH-containing ligands. To test this hypothesis, we used the povme software to calculate the volume of the pocket throughout the MD trajectory, and in numerous crystal structures. Figure 3A displays the volume of the HIP UPPS simulation binding pocket calculated for each selected frame along the time-course of the simulation, as well as a time-averaged size along the simulation, and the size of selected crystal structures. As can be seen, conformational states that show pocket volume close to the one observed in the *holo* crystal structure (2E98) are rarely sampled in the simulation of the *apo* form of UPPS. The crystal structure 2E99 is bound to another bisphosphonate BPH-608 (11) and also has a large pocket size, 873 Å^3^. Although protein coordinates from the crystal structure, 2E98, were used to build our UPPS model, when simulated in the absence of ligands, the pocket volume decreases significantly after the initial equilibration steps and never returns to its initial value during the production phase. Interestingly, at approximately 12 ns, the pocket widens to 939 Å^3^ and reaches within 100 Å^3^ of the size observed in the crystal structure (Figure 3A). In order to evaluate the influence of the protein pocket size on the docking results, the frame with the largest pocket was selected and used for further calculations. Docking of the BPH compounds to the selected structure showed a very good agreement with the experimental pIC_50_ values, with a correlation coefficient of −0.8 between the docking score and pIC_50_ (Figure S3). This represents a significant improvement when compared to the results obtained from the crystal structure 2E98 (correlation coefficient of −0.5). We partially attribute this improvement to the opening of the bisphosphonate binding region in Site 1. In the 2E98 crystal structure, the positively charged residues HIS43 and ARG77 are in close proximity. This region widens in the largest MD-derived structure, minimizing the scoring penalty for ligands with a positively charged N near the positively charged region of the protein, such as BPH-641 and BPH-642 (the two lowest scoring ligands when docked against 2E98). This repulsive force is strong enough that both BPH-641 and BPH-642 take on unexpected poses with poor scores (Figures S1 and S4). The wider bisphosphonate binding region is thus able to accommodate a larger variety of bisphosphonate containing ligands.

**Figure 4:**
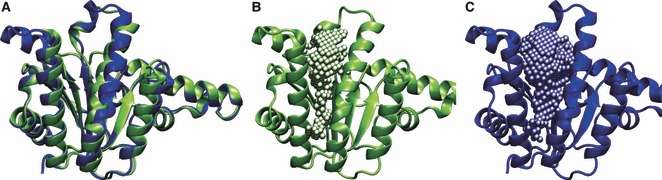
(A) The *apo* crystal structure in green and the bisphosphonate-bound crystal structure in blue. (B) The *apo* crystal structure with 1 Å spheres filling the active site pocket. (C) The bisphosphonate-bound crystal structure with 1 Å spheres filling the active site pocket. Note the significantly larger pocket size in the bisphosphonate-bound structure when compared to the *apo* crystal structure.

To help confirm the nature of this pocket closure on ligand removal (or expansion on ligand binding), we crystallized the *E. coli* UPPS in the ligand-free form. Full crystallographic data and structure refinement details are given in Table 1, and a comparison of this structure (PDB ID code 3QAS) with that of the bisphosphonate-containing species (PDB ID code 2E98) is shown in Figure 4. Crystal structures of the ligand-bound and *apo* form of UPPS reveal that a major pocket closure occurs upon ligand removal, from 1032 to 432 Å^3^ and is in good agreement with the time-averaged pocket volume (332 Å^3^) of the *apo* form obtained from the MD simulation.

**Chart 1:**
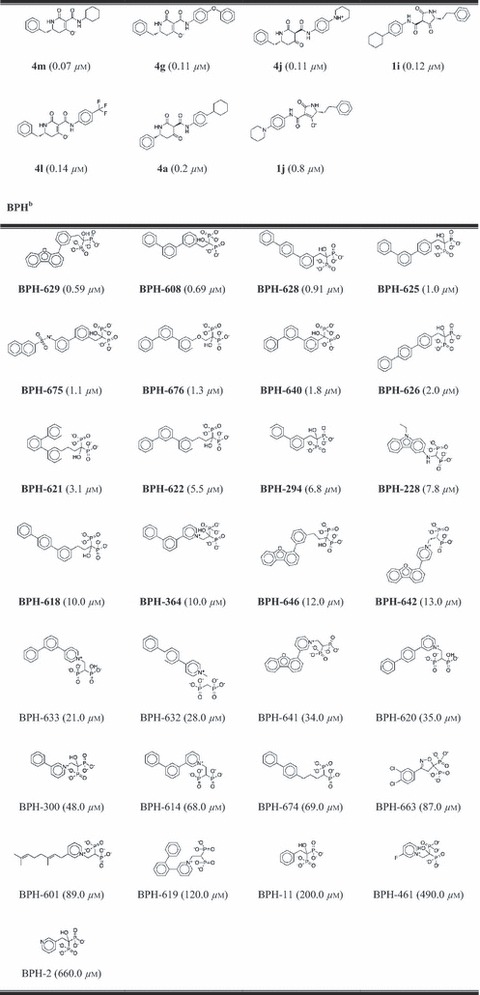
Undecaprenyl pyrophosphate synthase inhibitors. IC_50_ values shown in parentheses. ^a^Peukert 2008, (*S. pneumoniae* UPPS tested). ^b^Guo 2007, (*E. coli* UPPS tested).

To further investigate the binding mode of tetramic acid and dihydropyridin-2-one-3-carboxamide inhibitors, molecules **1i**, **1j**, **4a**, **4g**, **4j**, **4l,** and **4m** (Chart 1) were docked into the five most representative MD structures. Interestingly, only docking into the most representative member of the fourth cluster generated ligand poses similar to the one observed for the FPP natural substrate (Figure 2D). Unlike the results obtained from the crystal structure 2E98, all docked tetramic acid and dihydropyridin-2-one-3-carboxamide inhibitors reproduced the binding mode of the substrate FPP (Figure 2D). Because our bisphosphonates bind to structures with open active sites, we wanted to know whether these compounds, which were designed using a pharmacophore hypothesis of FPP binding, bind to the same size pockets as FPP-bound or *apo* crystal structures. Four crystal structures are described by Guo *et al.* (8) which have IPP or FSPP, an FPP analogue, in the active site. These structures had active site volumes ranging from 295 to 330 Å^3^, which is similar to the calculated volume of the fourth cluster, 377 Å^3^. This indicates that tetramic acids and dihydropyridine inhibitors, as well as the natural substrates, bind to more closed forms of the enzyme, similar to the *apo* state, while bisphosphonates bind to an open form.

**Figure 5:**
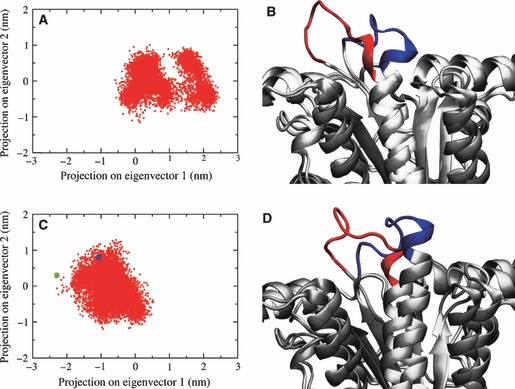
Principal component analyses (PCA). (A) Principal component analysis (PCA) calculated from the HID43 trajectory. (B) The extreme conformations of the flexible loop (residues 72–82) in HID43 are shown in red and blue. (C) PCA calculated from the HIP43 trajectory. The green circle indicates the largest conformation, and the blue circle indicates cluster 4. (D) The extreme conformations of the flexible loop (residues 72–82) in HIP43 are shown in red and blue.

As the conformational states that bind tetramic acid and dihydropyridine inhibitors are sparsely populated, and those that bind bisphosphonates are rarely sampled in our MD simulations, our results suggest that a population shift mechanism (30) may play an important role in changing the equilibrium towards other conformations upon inhibitor binding. Furthermore, it appears that the active site of the *apo* structure opens and expands considerably upon binding of bisphosphonate ligands, shifting the population of UPPS enzymes to a markedly different conformation. To further investigate this population shift, we plotted the principal components (PC) of our HIP trajectory (Figure 5C) and highlighted both the BPH binding structure (green) and tetramic acid and dihydropyridine binding structure (blue). Principal component analyses break the complex motions of molecular dynamics simulations into just a few variables. The two eigenvectors shown are the principal components of motion that account for the most motion. In the event of inhibitor binding, the PC results suggest a shift away from the center of the most highly sampled area of the *apo* MD simulation. Therefore, our results indicate that structurally diverse inhibitors recognize a specific set of conformational states of the receptor, which can vary significantly between families of ligands.

**Scheme 1:**
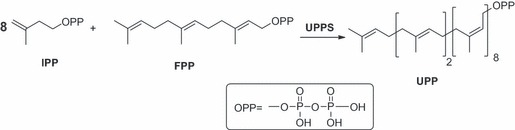
Condensation reaction of IPP and farnesyl pyrophosphate by undecaprenyl pyrophosphate synthase.

### Breathing motion of the catalytic pocket

The most dynamic region of UPPS is the loop comprising residues 72–82. Besides the terminals, the root mean square fluctuation (RMSF) of this loop region is by far the dominant feature in Figure S5 for both the HID43 and HIP43 simulations. The RMSF plots also reveal that the HIP43 simulations show a slightly more flexible protein, especially the helices as defined by residues 80–100 and 120–140. One of the highly populated conformational states of HID43 shows ARG77 deep inside the UPPS active site. Similar conformations were not observed in the HIP43 simulation. This result suggests that differences in electrostatic interactions, originating from different protonation states of active site HIS43, may affect the loop dynamics and the motion of the catalytically important residues on the flexible loop. It has been proposed that ARG77 plays an important role in the catalytic mechanism by helping transfer the pyrophosphate group from one substrate to another in the active site (5). Kinetic studies have shown a 1000-fold decrease in activity when ARG77 is mutated to ALA77 (7). Our MD trajectories revealed that ARG77 more extensively samples regions between the first and second sites in the HID43 than in the HIP43 simulations. This behavior can be attributed to the difference in electrostatic forces originating from the doubly protonated HIP43, which prevents this movement. The flexible loop in the HID43 simulation shows substantially more movement into and out of the pocket. The effect of the different protonation states of HIS43 on the dynamic behavior of UPPS can also be seen in Figure 5. The projection of the trajectories onto the first two eigenvectors calculated from principal component analysis reveals that each system clearly samples different regions of the conformational space. This result may have catalytic implications, as it suggests that ARG77 is only able to successfully transfer the pyrophosphate group between the two sites when HIS43 is singly protonated. Additionally, the imidazole group of HIS43 interacts directly with the pyrophosphate, and experimental results have shown that a 1000-fold decrease in catalytic activity is observed when this residue is mutated to ALA43. These results support the hypothesis proposed by Chang *et al.* (6) who suggest the initial binding of FPP is encouraged by the protonated imidazole group followed by proton donation from HIS43 to FPP, inducing changes in the dynamic behavior of the flexible loop.

## Conclusions

In this work, we performed long MD simulations, crystallography, docking studies, and pocket volume analysis on UPPS to investigate its dynamic behavior and the influence of protein flexibility on the design of UPPS inhibitors. We combined virtual screening procedures with MD simulations to incorporate protein dynamics into the drug design effort (31–34). Our MD simulations showed that UPPS is a highly flexible system, displaying very mobile active site pockets. Our results suggest that different classes of inhibitor molecules may recognize different ‘*active conformational states,’* some of which can be sparsely populated in the *apo* enzyme. We identified rare conformations of UPPS with expanded pocket volumes from an MD simulation that primarily sampled much smaller pockets. Moreover, we observed that the poses of co-crystallized bisphosphonate inhibitors can only be reproduced, with docking, when rarely sampled states with expanded pockets that exhibit conformations similar to bisphosphonate UPPS co-crystal structures are considered. Conversely, other inhibitor classes that mimic FPP binding require a conformation that is less expanded. Proper identification of the conformational state that specific inhibitors bind is thus important for structure-based drug design in UPPS. We also described the first complete *apo E. coli* UPPS structure with an intact active site. We observed an excellent agreement between the calculated average pocket volume from the *apo* enzyme simulation and the newly solved crystal structure. Since the successful design of new UPPS inhibitors is complicated by the intrinsic dynamic behavior of the receptor, the characterization and identification of these sparsely populated, but extremely relevant, *active conformational states* are of key importance in virtual screening efforts.

## Abbreviations

UPPS: Undecaprenyl pyrophosphate synthase
MD: molecular dynamics
IPP: isopentenyl diphosphate
FPP: farnesyl pyrophosphate
HTS: high throughput screens
BPH: bisphosphonate
FSPP: S-*thiolo*-farnesyl diphosphate
PC: principal components
PCA: principal component analysis
RMSD: root mean square deviation
RMSF: root mean square fluctuation

## Supporting Information

Figure S1 The 2E98 crystal structure, (A) Monomer A is represented in cartoon. bisphosphonate binding Sites 1, 2, 3, and 4 are represented in surface in red, gray, white, and brown respectively. (B) Bisphosphonate binding site 1 interaction map with labeled residues. Residues have light-green carbons, BPH-629 has gray carbons. Figure S2. RMSD of the Trajectories. Blue isthe HIP43 simulation, and red is the HID43 simulation – both show stability throughout the MD run. Figure S3. Scatter plot of pIC50 and predicted free energy of binding by GLIDE for BPH compounds. Figure S4. Poses of BPH-629 compared with BPH-641 docked against (A) the 2E98 crystal structure, and (B) the largest MD-derived structure. Figure S5. Root mean square fluctuation (RMSF) calculated for each residue in the MD simulations of HID43 (red) and HIP43 (blue) systems.

Appendix S1 Information on how to obtain and view the structurewith the largest active site from MD simulations.
