# Supplementary material for: Applying Molecular Dynamics Simulations to Identify Rarely Sampled Ligand-bound Conformational States of Undecaprenyl Pyrophosphate Synthase, an Antibacterial Target
Source: Chem Biol Drug Des. 2011 Jun;77(6):412–20. doi: 10.1111/j.1747-0285.2011.01101.x (PMC3095679; doi:10.1111/j.1747-0285.2011.01101.x)
Supplement: Figure S1 — The 2E98 crystal structure, (A) Monomer A is represented in cartoon. bisphosphonate binding Sites 1, 2, 3, and 4 are represented in surface in red, gray, white, and brown respectively. (B) Bisphosphonate binding site 1 interaction map with labeled residues. Residues have light-green carbons, BPH-629 has gray carbons. Figure S2. RMSD of the Trajectories. Blue isthe HIP43 simulation, and red is the HID43 simulation – both show stability throughout the MD run. Figure S3. Scatter plot of pIC50 and predicted free energy of binding by GLIDE for BPH compounds. Figure S4. Poses of BPH-629 compared with BPH-641 docked against (A) the 2E98 crystal structure, and (B) the largest MD-derived structure. Figure S5. Root mean square fluctuation (RMSF) calculated for each residue in the MD simulations of HID43 (red) and HIP43 (blue) systems. [file cbdd0077-0412-SD1.pdf]

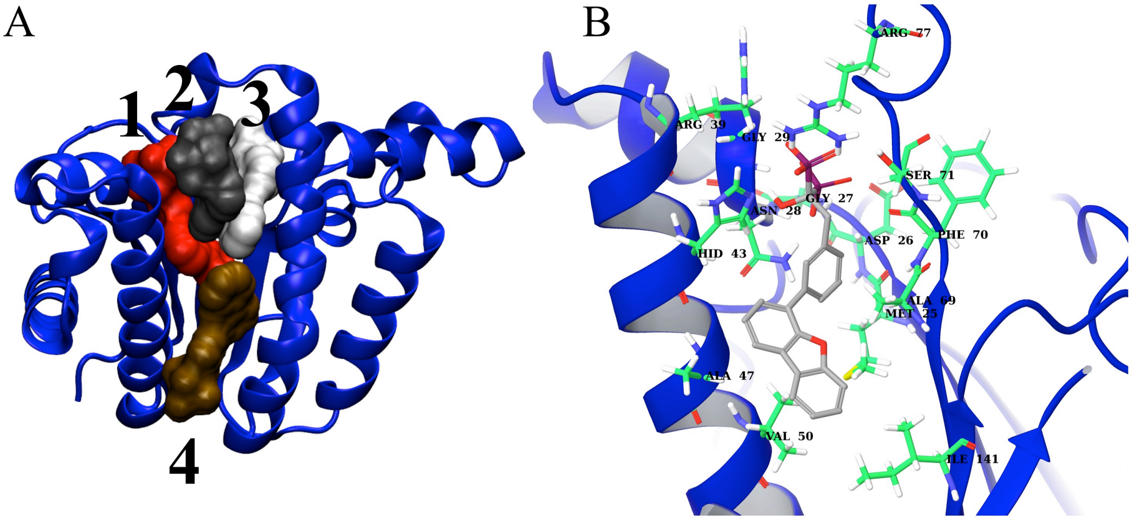

Figure S1. The 2E98 crystal structure, (A) Monomer A is represented in cartoon. bisphosphonate binding sites 1, 2, 3, and 4 are represented in surface in red, gray, white and brown respectively. (B) bisphosphonate binding site 1 interaction map with labeled residues. Residues have light green carbons, BPH-629 has gray carbons.

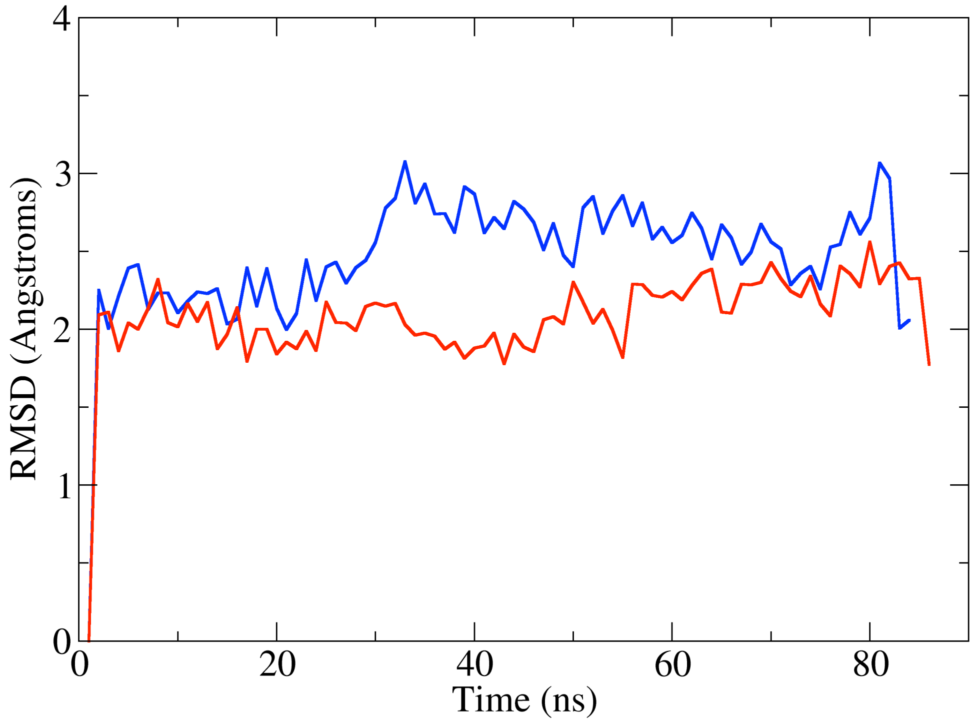

Figure S2: RMSD of the Trajectories. HIP43 (blue) simulation and HID43 (red) simulation, both show stability throughout the MD run.

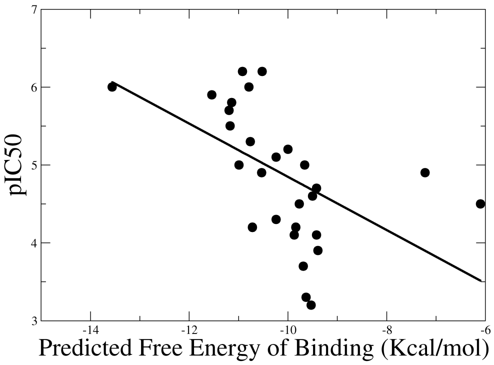

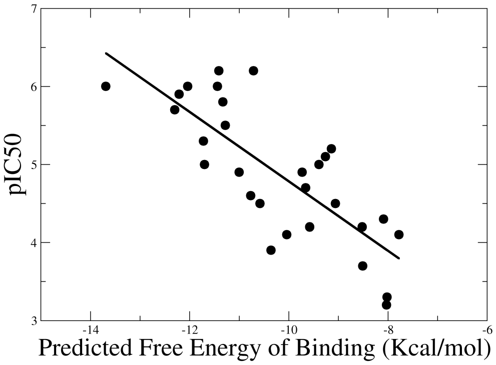

Figure S3: Scatter plot of pIC_{50} and predicted free energy of binding by GLIDE for BPH compounds. On the left is the 2E98 crystal structure docking results and linear regression line (R=-.52) and on the right is the largest structure from the MD simulation and linear regression line (R=-.79).

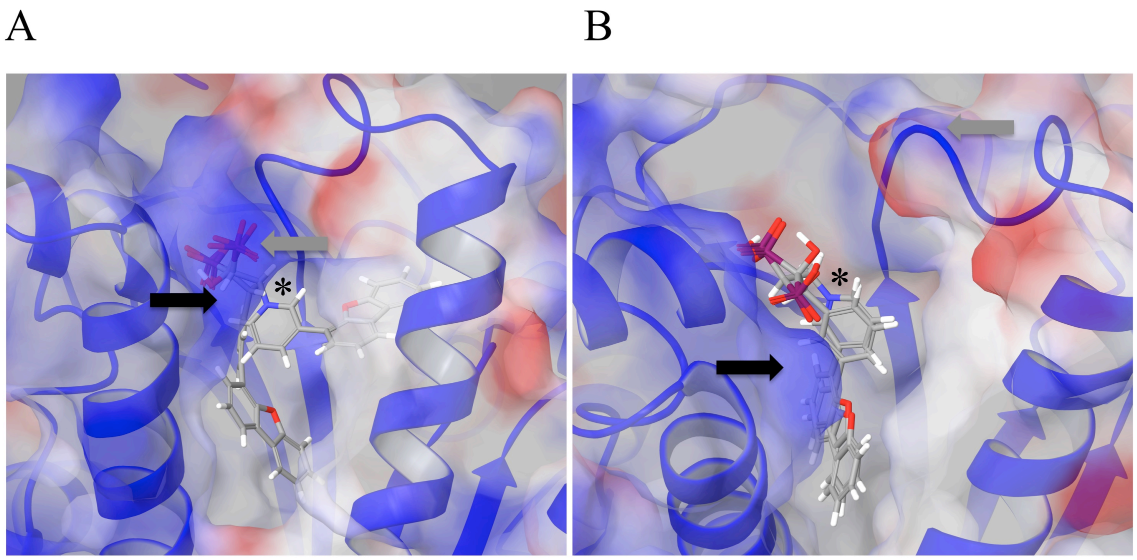

Figure S4. Poses of BPH-629 compared with BPH-641 docked against (A) the 2E98 crystal structure, and (B) the largest MD derived structure. The protein backbone is shown in cartoon representation with a transparent electrostatic surface representation (blue is positive and red negative). The black arrow points to HIS43, the gray arrow points to ARG77, the * denotes the nitrogen in BPH-641, ligands are represented in stick form with standard atom coloring. Note the narrow positively charged bisphosphonate binding region in the 2E98 structure and the unexpected pose generated by GLIDE for BPH-641.

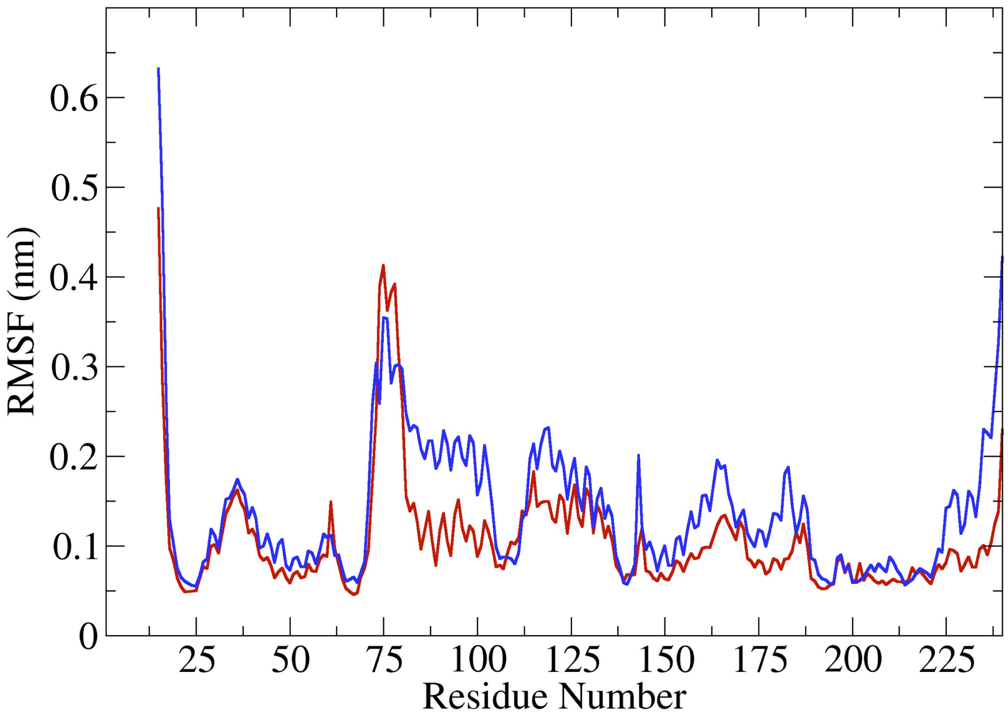

Figure S5. Root mean square fluctuation (RMSF) calculated for each residue in the MD simulations of HID43 (red) and HIP43 (blue) systems.

Complete Ref 19 D. Case, T. Darden, T. Cheatham, III, C. Simmerling, J. Wang, R. Duke, R. Luo, M. Crowley, R. C. Walker, W. Zhang, K. Merz, B. Wang, S. Hayik, A. Roitberg, G. Seabra, I. Kolossvry, K. Wong, F. Paesani, J. Vanicek, X. Wu, S. Brozell, T. Steinbrecher, H. Gohlke, L. Yang, C. Tan, J. Mongan, V. Hornak, G. Cui, D. Mathews, M. Seetin, C. Sagui, V. Babin, and P. Kollman, "AMBER 10," University of California, San Francisco, 2008.

Pocket Volume Calculation Code

The POVME software is available at http://www.nbcr.net/POVME/.
