# Supplementary material for: Applying Molecular Dynamics Simulations to Identify Rarely Sampled Ligand-bound Conformational States of Undecaprenyl Pyrophosphate Synthase, an Antibacterial Target
Source: Chem Biol Drug Des. 2011 Jun;77(6):412–20. doi: 10.1111/j.1747-0285.2011.01101.x (PMC3095679; doi:10.1111/j.1747-0285.2011.01101.x)
Supplement: Appendix S1 — Information on how to obtain and view the structurewith the largest active site from MD simulations. [file cbdd0077-0412-SD1.doc]

Appendix S1.

The structure with the largest active site from our MD simulations can be downloaded here in .pdb format:

This file can be opened with any molecular visualization software that opens .pdb files like VMD.  Information on how to obtain and use VMD is available here:

<http://www.ks.uiuc.edu/Research/vmd/> 
